# Acute Hepatitis due to Primary Human Immunodeficiency Virus Infection

**DOI:** 10.1093/ofid/ofae170

**Published:** 2024-03-22

**Authors:** Eric I Elliott, Daisy Smith, Jonathan Lipscomb, Bubu Banini, Lindsay Meurer, Thomas H Vanderford, Jeffrey A Johnson, Dhanpat Jain, Amit Achhra

**Affiliations:** Section of Infectious Disease, Department of Internal Medicine, Yale University School of Medicine, New Haven, Connecticut, USA; The DESA Group, Inc., Columbia, South Carolina, USA; HIV Laboratory Division of HIV Prevention, Centers for Disease Control and Prevention, Atlanta, Georgia, USA; Section of Digestive Diseases, Department of Internal Medicine, Yale University School of Medicine, New Haven, Connecticut, USA; Division of Digestive and Liver Diseases, University of Texas Southwestern Medical Center, Dallas, Texas, USA; HIV Laboratory, Division of HIV Prevention, Centers for Disease Control and Prevention, Atlanta, Georgia, USA; HIV Laboratory, Division of HIV Prevention, Centers for Disease Control and Prevention, Atlanta, Georgia, USA; Department of Pathology, Yale University School of Medicine, New Haven, Connecticut, USA; Section of Infectious Disease, Department of Internal Medicine, Yale University School of Medicine, New Haven, Connecticut, USA

**Keywords:** acute hepatitis, human immunodeficiency virus, acute retroviral syndrome, acute HIV infection, primary HIV infection, liver, histopathology, immunohistochemistry, immunofluorescence, proviral DNA

## Abstract

The acute retroviral syndrome may present with diverse systemic manifestations and laboratory abnormalities. Here we present a rare case of primary human immunodeficiency virus (HIV) infection causing severe acute hepatitis. Liver histopathology demonstrated a pattern of lymphocytic inflammation consistent with acute hepatitis, high levels of HIV proviral DNA were detected within liver tissue, and immunofluorescence showed HIV p24 antigen within immune and parenchymal cells including hepatocytes. We review the literature pertaining to HIV infection of cell compartments within the liver and discuss the implications for HIV-associated acute liver disease.

Primary human immunodeficiency virus infection (PHI) may manifest as acute retroviral syndrome (ARS)—a mononucleosis-like illness characterized by fever, diaphoresis, malaise, fatigue, anorexia, and lymphadenopathy [1]. Patients with PHI presenting with ARS have high viral load (VL), low CD4 count, and inflammation in multiple tissue compartments [2]. As ARS is usually a self-limited condition, it is unclear whether acute organ-specific inflammatory manifestations have lasting physiologic consequences [2]. However, it is postulated that early initiation of antiretroviral therapy (ART) prevents adverse long-term clinical outcomes by limiting development of the human immunodeficiency virus infection (HIV) reservoir in these tissue compartments [3, 4]. While mild liver function testing (LFT) abnormalities are common in both primary and chronic HIV infection [5, 6], it is exceedingly rare for patients with PHI to present with symptomatic hepatitis [7]. Furthermore, the histopathologic features and the localization of HIV within the liver in the setting of HIV-associated acute hepatitis have not been characterized in the literature. Here we report a case of severe acute hepatitis in a patient with ARS with no plausible alternative infectious, inflammatory, autoimmune, or genetic cause. We describe the histologic features of the liver biopsy, demonstrate remarkably high levels of HIV proviral DNA within liver tissue by polymerase chain reaction (PCR), and detect the presence of HIV p24 antigen within multiple cell types including hepatocytes.

## CASE PRESENTATION

A man in his early 20s presented to the emergency department (ED) with 2 weeks of fevers, headache, sore throat, myalgias, and malaise. He also noted 1 week of nausea and diarrhea and 2 days of a pruritic rash on the chest, back, and hands. Further history revealed he had returned to the United States the day preceding symptom onset after 2 weeks of travel to South America. He endorsed condomless intercourse with a male partner 1 month prior to presentation. He received a tattoo 3 weeks prior to presentation. Prior to presenting to our hospital's ED, he was prescribed amoxicillin-clavulanate at an outside institution, which he self-discontinued after 3 days due to lack of improvement. In addition, he reported taking acetaminophen for fevers and myalgias without exceeding 2 g daily. He endorsed sporadic alcohol use of no more than 4 drinks on 1 or 2 occasions monthly. He denied recreational drug use including injection drug use.

On his initial ED presentation, his temperature was 37.6°C (100.1°F), blood pressure 127/73 mm Hg, pulse 108 beats per minute (bpm), respiratory rate 18 breaths per minute, and oxygen saturation 99%. Physical examination demonstrated generalized abdominal tenderness without guarding or rebound. A papular, erythematous rash was noted on his dorsal hands and abdomen. Laboratory investigations were notable for white blood cell (WBC) count of 2.4 × 10^3^ cells/μL (reference range: 4–11 × 10^3^ cells/μL), absolute lymphocyte count (ALC) of 0.42 × 10^3^ cells/μL (reference range: 0.6–3.7 × 10^3^ cells/μL) with 2.6% atypical lymphocytes, and mildly elevated transaminases with aspartate aminotransferase (AST) of 88 U/L (reference range: 10–35 U/L) and alanine aminotransferase (ALT) of 75 U/L (reference range: 9–59 U/L). Complete results of hematology and chemistry testing are shown in Table 1. He was discharged from the ED while a preliminary infectious workup was pending (Table 2). A fourth-generation screen for HIV was positive, the HIV-1 and -2 differentiation assay was negative for HIV-1 or -2 antibodies, and HIV-1 PCR was positive with >10 million copies/mL (Table 2).

**Table 1.** Basic Laboratory Studies

Laboratory Variable	Reference Range	Day 0	Day 3	Day 6	Day 7	Day 8
Hematology						
Hemoglobin (g/dL)	13.2–17.1	14.3	15.4	14.2	13.0	…
Hematocrit (%)	38.5–50.0	42.4	45.0	41.6	37.7	…
Platelets (cells × 10^3^/μL)	150–420	156	190	202	200	…
WBC (cells × 10^3^/μL)	4.0–11.0	2.4	3.2	4.0	8.0	…
Neutrophils (cells × 10^3^/μL)	2–7.6	1.87	2.3	1.32	0.96	…
Monocytes (cells × 10^3^/μL)	0–1.0	0.11	0.22	0.92	1.20	…
Eosinophils (cells × 10^3^/μL)	0–1.0	0.00	0.00	0.00	0.00	…
Lymphocytes (cells × 10^3^/μL)	0.6–3.7	0.42	0.61	1.76	5.84	…
Atypical lymphocytes (%)	0–1.0	2.6	8.0	13.0	34.0	…
Bands (%)	0–10.0%	5.3	14.0	1.0	2.0	…
Chemistry						
Sodium (mmol/L)	136–144	135	135	131	133	134
Potassium (mmol/L)	3.3–5.3	4.1	3.7	3.5	3.7	4.2
Chloride (mmol/L)	98–107	102	103	95	98	102
CO_2_ (mmol/L)	20–30	21	21	25	23	23
Glucose (mg/dL)	70–100	105	98	108	94	99
BUN (mg/dL)	6–20	7	5	8	5	5
Creatinine (mg/dL)	0.40–1.30	0.65	0.68	0.66	0.64	0.52
Calcium (mg/dL)	8.8–10.2	8.5	8.2	8.4	7.8	8.0
Total bilirubin (mg/dL)	≤1.2	0.4	0.7	0.9	0.7	0.6
Direct bilirubin (mg/dL)	≤0.3	<0.2	0.2	…	…	…
Alkaline phosphatase (U/L)	9–122	111	307	490	429	395
Alanine aminotransferase (U/L)	9–59	75	473	818	821	838
Aspartate aminotransferase (U/L)	10–35	88	534	864	627	633
GGT (U/L)	<48	…	306	…	…	…
Total protein (g/dL)	6.6–8.7	6.5	6.4	6.4	5.8	5.8
Albumin (g/dL)	3.6–4.9	4.0	3.7	3.7	3.1	3.3
Globulin (g/dL)	2.3–3.5	2.5	2.7	2.7	2.7	2.5
Prothrombin time (sec)	9.6–12.3	…	11.4	10.9	11.3	11.4
INR	0.87–1.14	…	1.05	1.00	1.04	1.05
Lipase (U/L)	11–55	24	20	…	…	…
Creatine kinase (U/L)	11–204	…	72	…	…	…
hs-CRP (mg/L)	<1.0	…	24.9	…	…	…

Abbreviations: BUN, blood urea nitrogen; CO_2_, carbon dioxide; GGT, γ-glutamyl transferase; hs-CRP, high-sensitivity C-reactive protein; INR, international normalized ratio; WBC, white blood cell.

**Table 2.** Laboratory Diagnostic Testing

Variable	Reference Range	Result	Day(s) of Collection
Blood			
HIV-1 and -2 Ab/HIV-1 Ag screen	Negative	Positive	0
HIV-1 antibody confirmation	Negative	Negative	1
HIV-2 antibody confirmation	Negative	Negative	1
HIV-1 RT-PCR (copies/mL)	Not detected	>10 000 000	1
HIV-1 RT-PCR (log_10_ copies/mL)	Not detected	>7.00	1
CD4 (cells/μL)	466–1608	148	4
CD4 (%)	29.33–59.86	7.64	4
*Treponema pallidum* Ab	Nonreactive	Nonreactive	3
Blood cultures, 2 sets	No growth	No growth	0, 4, 10
Lyme Ab	≤0.90 LI	0.48	3
*Anaplasma phagocytophilum* PCR	Not detected	Not detected	3
*Ehrlichia* PCR	Not detected	Not detected	3
*Babesia* smear	Negative	Negative	3
Malaria smear	Negative	Negative	0
QBC for blood parasite	Negative	Negative	0, 3
Hepatitis A IgG	Negative	Positive	3
Hepatitis A IgM	Negative	Negative	3
Hepatitis B core Ab, total	Negative	Negative	3
Hepatitis B core IgM	Negative	Negative	6
Hepatitis B surface Ab (mIU/mL)	≥ 12	69.26	3
Hepatitis B surface Ag	Negative	Negative	3
Hepatitis B PCR, blood	Not detected	Not detected	7
Hepatitis C Ab	Negative	Negative	3
Hepatitis C PCR, blood	Not detected	Not detected	3
Hepatitis E IgM	Not detected	Not detected	6
Hepatitis E IgG	Not detected	Not detected	6
Hepatitis E PCR, blood	Not detected	Not detected	7
HSV-1/2 PCR, blood	Not detected	Not detected	7
EBV heterophile antibodies/monospot	Negative	Negative	3
EBNA-1 IgG (U/mL)	≤17.9	200	12
EBV VCA IgM (U/mL)	≤35.9	34.6	12
EBV VCA IgG (U/mL)	≤17.9	>750	12
EBV PCR, blood	Not detected	Not detected	6
CMV PCR, blood	Not detected	Not detected	6
Toxoplasmosis IgM (OD ratio)	≤0.90	0.76	3
Toxoplasmosis IgG (OD ratio)	≤0.90	0.18	3
Dengue fever virus IgM	<1.65	0.93	7
Dengue fever virus IgG	<0.80	0.12	7
*Leptospira* IgM	Negative	Negative	8
*Leptospira* PCR	Not detected	Not detected	7
QuantiFERON-TB Gold	Negative	Negative	18
(1→3)-β-D-glucan (Fungitell) (pg/mL)	<60	<31, negative	6
Cryptococcal Ag	Negative	Negative	10
Acetaminophen (μg/mL)	10–30	<5	4
ANA	<1:80	<1:80	6
Smooth muscle (F-actin) IgG (U)	<20	8	6
IgG (mg/dL)	700–1600	995	7
Ceruloplasmin (mg/dL)	18–51	26	7
Iron (μg/dL)	59–158	57	7
Ferritin (ng/mL)	30–400	8705	7
Urine			
Histoplasma galactomannan Ag (ng/mL)	<0.2	<0.2	6
Urine 9-drug toxicology panel	Negative	Negative	4
Nasopharyngeal			
SARS-CoV-2 RNA	Negative	Negative	0, 3
Influenza A and B/RSV	Negative	Negative	3

Abbreviations: Ab, antibody; Ag, antigen; ANA, antinuclear antibody; CMV, cytomegalovirus; EBNA, Epstein-Barr nuclear antigen; EBV, Epstein-Barr virus; HIV, human immunodeficiency virus; HSV, herpes simplex virus; IgG, immunoglobulin G; IgM, immunoglobulin M; LI, Lyme index; OD, optical density; PCR, polymerase chain reaction; QBC, quantitative buffy coat; RSV, respiratory syncytial virus; RT-PCR, reverse-transcription polymerase chain reaction; SARS-CoV-2, severe acute respiratory syndrome coronavirus 2; VCA, viral capsid antigen.

**Figure 1.**
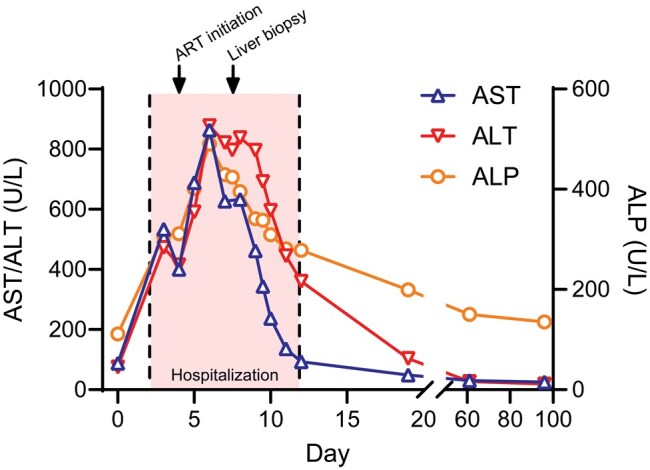
Liver enzyme trend. On day 0, the patient first presented to the emergency department. The patient returned and was hospitalized from days 3 through 12 (shaded area). Antiretroviral therapy was initiated on day 5 and liver biopsy was performed on day 8 (arrows). Abbreviations: ALP, alkaline phosphatase; ALT, alanine aminotransferase; ART, antiretroviral therapy; AST, aspartate aminotransferase.

Three days later, prompted by a telephone call, he returned to the ED for further evaluation. He became febrile to 39.3°C (102.8°F), and ceftriaxone and doxycycline were started prior to infectious disease evaluation. He remained tachycardic at 122 bpm and normotensive at 123/74 mm Hg. Examination revealed extensive posterior pharyngeal erythema without exudate or petechiae, cervical and submandibular lymphadenopathy, epigastric tenderness, and a more apparent rash. The patient did not demonstrate any evidence of encephalopathy. Laboratory results were notable for WBC count 3.2 ×10^3^ cells/μL, ALC of 0.61 × 10^3^ cells/μL, 8% atypical lymphocytes, and 14% bands (Table 1). AST had increased to 534 U/L, ALT to 473 U/L, and alkaline phosphatase (ALP) to 307 U/L, and γ-glutamyl transferase was 306 U/L (reference range: <48 U/L) (Table 1, Figure 1). Initial CD4 T-cell count was 148 cells/μL (reference range: 466–1608 cells/μL) (Table 2). During hospitalization, the liver enzymes continued to rise with a peak AST of 864 U/L, ALT of 838 U/L, and ALP of 490 U/L (Table 1, Figure 1). However, total and direct bilirubin, albumin, prothrombin time, and international normalized ratio remained within normal limits. Extensive laboratory testing was negative for alternative acute infectious and noninfectious etiologies of hepatitis including herpes simplex virus (HSV), Epstein-Barr virus (EBV), cytomegalovirus (CMV), hepatitis A, hepatitis B, hepatitis C, and hepatitis E (Table 2). Abdominal ultrasound with duplex demonstrated a heterogenous liver parenchyma without biliary or venous obstruction. Magnetic resonance cholangiopancreatography was unremarkable. On day 8, the patient underwent percutaneous liver biopsy to determine the cause of hepatitis.

**Figure 2.**
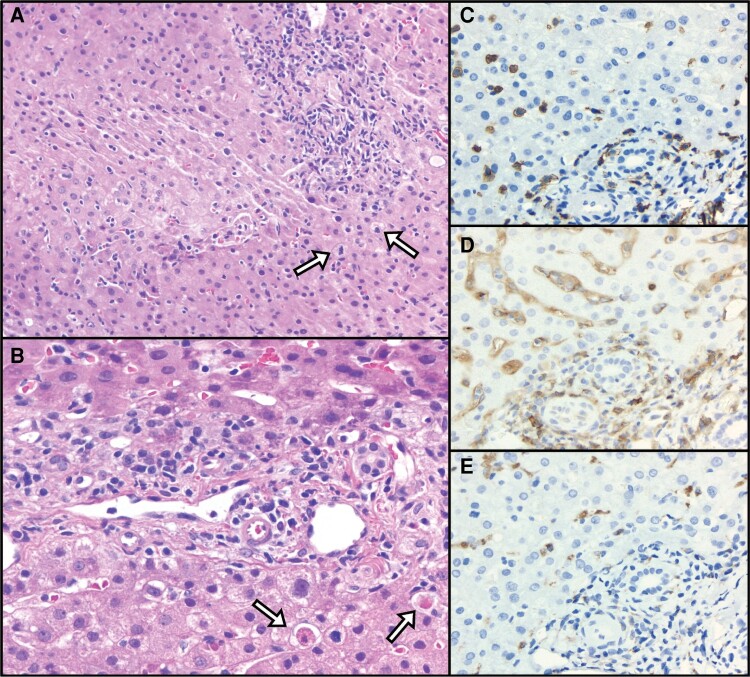
Liver histopathology demonstrating acute hepatitis. *A*, Liver biopsy showing a portal tract with moderate lymphomononuclear infiltrate, mild interface activity, increased lymphocytes in the hepatic sinusoids, and mitotic figures in hepatocytes (arrows) (hematoxylin and eosin [H&E] stain ×100). *B*, Medium-power view of a portal tract and periportal area showing the portal inflammation. Rare plasma cells can be seen admixed with lymphocytes. Few acidophil bodies are seen in the periportal area (arrows) (H&E stain ×200). *C–E*, Immunostains for CD8 (*C*) and CD4 (*D*) showing that most of the lymphocytes are CD8^+^ T cells, with few scattered CD4^+^ positive cells in the sinusoids and occasional CD4^+^ cells in the portal tract, while CD68 immunostaining (*E*) highlights sinusoidal Kupffer cells and portal macrophages (immunohistochemistry ×200).

**Figure 3.**
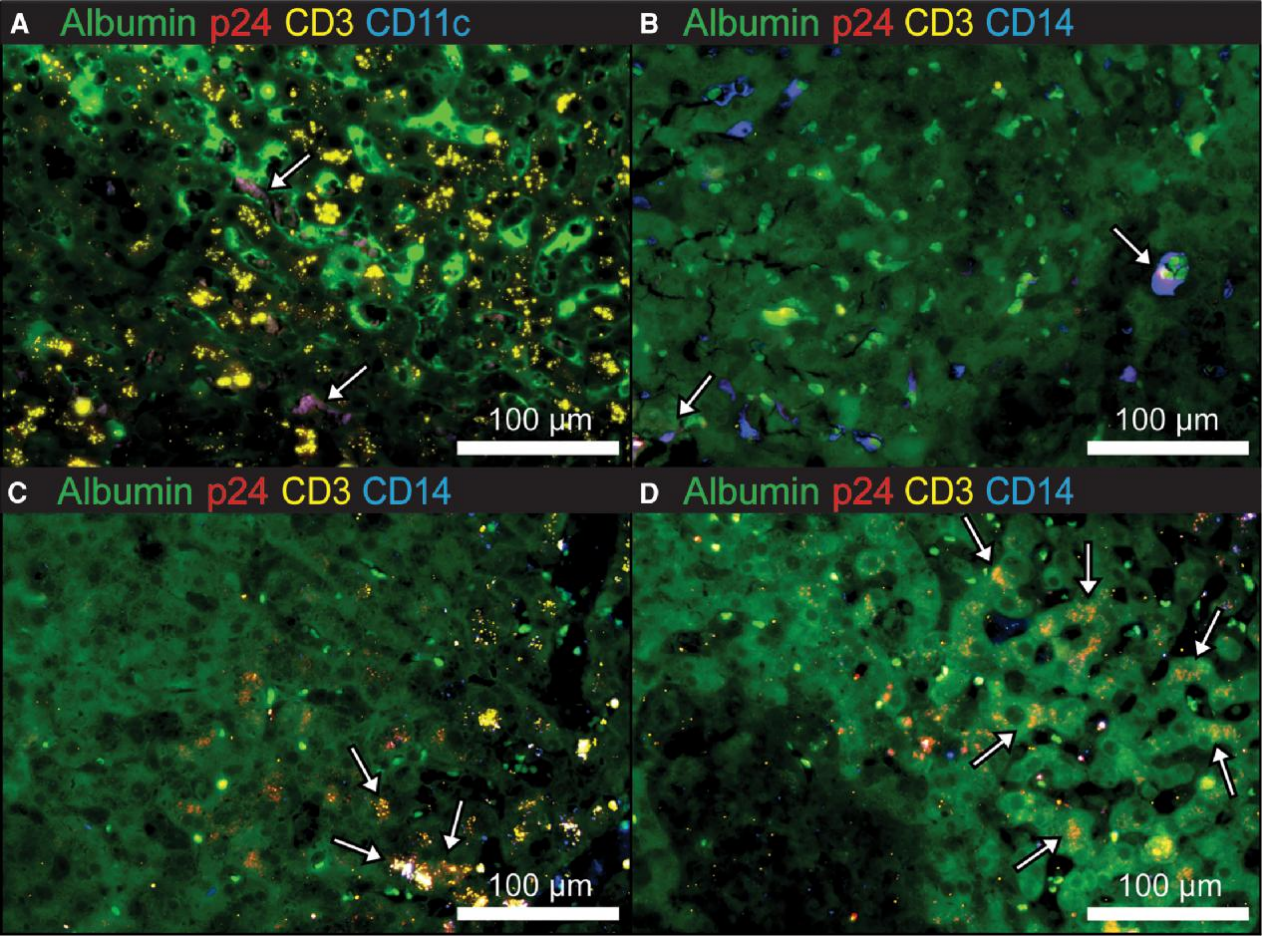
Immunofluorescence microscopy demonstrating human immunodeficiency virus (HIV) p24 antigen within liver tissue. *A*, Immunofluorescence microscopy of liver sections stained for HIV p24 (red), CD11c (blue), CD3 (yellow), and albumin (green) showing p24 co-localization with CD11c (arrows). *B–D*, Immunofluorescence microscopy of liver sections stained for HIV p24 (red), CD14 (blue), CD3 (yellow), and albumin (green) showing areas of co-localization (arrows) of p24 antigen with CD14 (*B*), CD3 (*C*), and albumin alone (*D*). All images obtained using ×20 objective.

Liver biopsy showed extensive lobular lymphomononuclear inflammation with predominantly CD8^+^ lymphocytes lacking cytologic atypia and inconsistent with lymphoma (Figure 2*A*). Plasma cells and CD20^+^ B cells were scant. Scattered acidophil bodies were noted (Figure 2*B*). The portal inflammation was similar, with mild lymphocytic infiltration predominantly composed of CD8^+^ lymphocytes (Figure 2*C* and 2*D*). There was no significant fibrosis, steatosis, cholestasis, ballooning degeneration, or hepatocytic necrosis. Marked sinusoidal lymphocytosis and mild Kupffer cell hyperplasia were noted (Figure 2*C* and 2*E*). Overall, the features were consistent with acute hepatitis with a differential diagnosis including viral infection, lymphoproliferative disorder, and drug-induced liver injury (DILI). Immunostains were negative for HSV, EBV, CMV, and adenovirus (not shown). Based on the clinical scenario, the liver specimens were further evaluated for the presence of HIV at the Centers for Disease Control and Prevention (CDC). DNA was extracted from liver tissue (QIAamp DNA FFPE Tissue Kit, Qiagen), further purified (Monarch PCR & DNA Cleanup Kit, New England Biolabs), and HIV *Int* was detected by a CDC laboratory–developed quantitative PCR and normalized to human RNase P. High amounts of proviral DNA (46–936 copies/100 cells) were detected in liver biopsy specimens from the patient (reference range: 1 copy per 10^4^–10^6^ peripheral blood mononuclear cells). The test for viral total nucleic acid (HIV-1 RNA and DNA) yielded equivalent amplification to that of proviral DNA testing alone and the predominant DNA presence was confirmed when DNase treatment of sample extract yielded little or no amplifiable HIV nucleic acid. Furthermore, abundant HIV p24 antigen was detected within liver sections by immunofluorescence microscopy and p24 co-localized with CD11c^+^ dendritic cells, CD14^+^ Kupffer cells, CD3^+^ T cells, and albumin-expressing hepatocytes (Figure 3).

The patient was started on ART with bictegravir, emtricitabine, and tenofovir alafenamide. His liver enzymes steadily improved and he was discharged from the hospital on day 12 (Figure 1). Two weeks later, his VL had decreased to 2550 copies/mL and CD4 count had recovered to 904 cells/μL (12.58%). At follow-up 6 weeks later, VL had further decreased to 73.5 copies/mL, CD4 remained stable at 804 cells/μL (30.45%), and LFT had completely normalized (Figure 1).

## DISCUSSION

Here we report a case of ARS presenting with severe acute hepatitis. PHI was confirmed by positive HIV antigen screening and PCR with negative initial antibody testing and subsequent seroconversion. The hepatitis was symptomatic with nausea and abdominal tenderness but was not associated with clinically apparent liver dysfunction. Transaminase elevations were severe (AST >15 times the upper limit of normal) without exceeding 1000 U/L, and they resolved rapidly with ART initiation. Although numerous epidemiological risk factors put him at risk for established infectious etiologies of hepatitis, exhaustive laboratory workup revealed no additional infections beyond HIV. While exposures to acetaminophen and amoxicillin-clavulanate preceded hepatitis onset, acetaminophen intake remained below 2 g daily and liver histopathology did not support DILI from either agent. Histopathologic examination of the liver biopsy confirmed acute hepatitis, liver tissue yielded an enormous burden of HIV proviral DNA, and HIV p24 antigen co-localized with dendritic cells, Kupffer cells, lymphocytes, and hepatocytes by immunofluorescence microscopy. Our analysis was limited by an inability to detect HIV RNA in the liver biopsy, suggesting compromised RNA stability in the specimens. Together, the clinical presentation and diagnostic findings provide definitive evidence of severe acute hepatitis due to PHI.

While mild transaminase elevations are apparent in nearly 16% of patients presenting with PHI without coinfections [6], overt hepatitis is exceedingly rare. Similar to our case, Abu-Heija and colleagues identified an HIV-seronegative patient with HIV VL >10 million copies/mL who developed transaminase elevations exceeding 1000 U/L [7]. Whereas our patient endorsed abdominal pain and nausea, their patient was asymptomatic but developed liver dysfunction as evidenced by coagulopathy on laboratory testing [7]. Many genetic, autoimmune, and infectious causes of hepatitis were carefully excluded; however, as liver biopsy was not performed there was not a direct examination of HIV presence within liver [7]. Therefore, together with our findings demonstrating abundant HIV in liver tissue, this case adds further evidence that acute HIV can directly cause severe acute hepatitis. Recognizing HIV as an etiology of acute hepatitis, we recommend that patients presenting with acute hepatitis and epidemiologic risk factors for HIV undergo thorough assessment of HIV status with blood p24 antigen testing and PCR when testing for more common etiologies is negative. Further expensive diagnostic testing, including invasive testing, may not be warranted for self-limited hepatitis in the setting of ARS improving on ART.

Yet, it is unintuitive how HIV contributes to hepatocellular injury. In vitro studies have yielded conflicting results and mixed conclusions regarding the potential for HIV to directly infect hepatocytes: Some report that hepatocytes express CD4 and support HIV infection [8, 9], others find that hepatocytes do not express CD4 yet remain susceptible to HIV infection [10–13], and still others demonstrate that HIV cannot infect hepatocytes or infects only with extremely low efficiency [14–16]. With uncertainty surrounding the contribution of direct infection of hepatocytes to HIV-associated liver pathology, other cell populations within liver tissue have also been examined. For instance, Kupffer cells appear susceptible to HIV infection, which sensitizes them to further inflammatory stimuli [17–19], and hepatic stellate cells reportedly express both CXCR4 and CCR5 to permit HIV infection in vitro [20], both providing a plausible indirect mechanism by which HIV induces hepatocellular injury.

Our findings, demonstrating for the first time that HIV is detected within hepatocytes during PHI in a patient with acute hepatitis, provide an impetus to further elucidate conditions and mechanisms required for direct HIV infection of hepatocytes. Furthermore, recent work suggesting hepatocytes are a reservoir for HIV in patients durably suppressed on ART [21] and prior phylogenetic evidence of HIV compartmentalization within the liver [22] underscore the importance of prompt initiation of ART to limit further establishment of a hepatic HIV reservoir. In this analysis, the ability to measure HIV-1 RNA–expressing cells was limited by an absence of amplifiable viral RNA, which may not have been preserved during specimen preparation. Whether limiting progression of the hepatocyte reservoir by early ART initiation curtails susceptibility of people with HIV to other forms of chronic liver disease warrants further investigation. Finally, recognizing that HIV may infect hepatocytes independent of traditional HIV co-receptors has implications for research aimed toward treating and curing HIV by pharmacologically blocking and genetically editing HIV co-receptors.

## Notes

***Author contributions.*** E. I. E. provided inpatient and outpatient infectious disease clinical care for the patient and constructed the manuscript. A. A. provided clinical care for the patient and reviewed all stages of the manuscript. B. B. and L. M. provided hepatology consultation for the patient. D. J. provided pathology interpretation and reviewed all stages of the manuscript. D. S., under supervision of J. A. J., provided HIV total nucleic acid real-time polymerase chain reaction detection and immunofluorescence images. J. L. and T. H. V. provided proviral DNA sequencing under supervision of J. A. J. All authors read and approved the final manuscript.

***Patient consent.*** Patient consent is not required due to anonymization of the case.

***Financial support.*** This work was supported by the National Institutes of Health (grant numbers T32 AI007517 and T32 HL007974 to E. I. E.).
